# **Autosuggestion: a cognitive process that empowers your brain?**

**DOI:** 10.1007/s00221-021-06265-8

**Published:** 2021-11-19

**Authors:** Kasia A. Myga, Esther Kuehn, Elena Azanon

**Affiliations:** 1 Faculty of Natural Sciences, Otto Von Guericke University Magdeburg, 39106 Magdeburg, Germany; 2 Department of Behavioral Neurology, Leibniz Institute for Neurobiology, 39118 Magdeburg, Germany; 3 Institute for Cognitive Neurology and Dementia Research (IKND), Otto-Von-Guericke University Magdeburg, 39120 Magdeburg, Germany; 4 Center for Behavioral Brain Sciences (CBBS) Magdeburg, 39120 Magdeburg, Germany; 5 Department of Neurology, Otto-Von-Guericke University, 39120 Magdeburg, Germany

**Keywords:** Autosuggestion, Top-down control, Therapy, Somatosensory systems

## Abstract

Autosuggestion is a cognitive process that is believed to enable control over one’s own cognitive and physiological states. Despite its potential importance for basic science and clinical applications, such as in rehabilitation, stress reduction, or pain therapy, the neurocognitive mechanisms and psychological concepts that underlie autosuggestion are poorly defined. Here, by reviewing empirical data on autosuggestion and related phenomena such as mental imagery, mental simulation, and suggestion, we offer a neurocognitive concept of autosuggestion. We argue that autosuggestion is characterized by three major factors: reinstantiation, reiteration, and volitional, active control over one’s own physiological states. We also propose that autosuggestion might involve the ‘overwriting’ of existing predictions or brain states that expect the most common (but not desired) outcome. We discuss potential experimental paradigms that could be used to study autosuggestion in the future, and discuss the strengths and weaknesses of current evidence. This review provides a first overview on how to define, experimentally induce, and study autosuggestion, which may facilitate its use in basic science and clinical practice.

## Introduction to the cognitive phenomenon of autosuggestion

The concept of autosuggestion is based on the captivating idea that an individual has control over widespread cognitive and physiological brain states. Autosuggestive techniques date back to the late nineteenth century when autosuggestion was introduced by Emile Coué. Since then, they are an integral part of our modern life. For example, a popular form of applied autosuggestive techniques are positive affirmations (i.e., statements of desired outcomes that people reiterate). Nevertheless, a systematic description of the cognitive and neural processes that underlie autosuggestion, similarities, and differences to existing concepts are scarce. Research questions such as ‘How can autosuggestion be defined in light of modern cognitive neuroscience?’ and ‘How can autosuggestion be experimentally induced in a laboratory setting?’ remain largely unanswered. This has consequences on the potential impact of autosuggestion techniques on a variety of scientific and clinical fields, for example in therapies for chronic pain or rehabilitation, which is still underexplored.

Here, we review evidence on autosuggestion and related phenomena, in particular mental imagery, mental simulation, suggestion (including placebo) and hypnosis, to disentangle these phenomena at a theoretical and practical level, and to identify and define cognitive features unique to autosuggestion. We describe experimental attempts used in the past to induce autosuggestion and outline some of their strengths and weaknesses. Evidence gathered here will help to make autosuggestion a future target for empirical research in cognitive neuroscience, subserving the development of evidence-based cognitive therapy in the mental health sector.

Whereas autosuggestion can be discussed in different contexts (Ludwig et al. 2014; Sari et al. 2017; Schlamann et al. 2010), here, we focus on the influence of autosuggestion on sensorimotor processing and perception of touch and pain processing. Sensorimotor systems are a suitable model for the investigation of precise sensory encoding principles that can be tested by using rigid and replicable experimental paradigms. Furthermore, the potential application of these insights to modify the perception of touch and pain makes it a particularly valuable target for basic and applied studies.

Autosuggestion is a process by which the implementation of an idea results in changes in perceptual and/or brain states, in the form of a so-called ‘self-administered suggestion. If such alterations in perceptual or brain states cannot be detected, according to our definition, autosuggestion did not take place. Self-induced suggestion differs from heterosuggestion, because the latter implies that suggestions are reinforced *by another person*, whereas those are reinforced *by the to-be-suggested person* in autosuggestion (see Box 1). We define autosuggestion as the *instantiation and reiteration of ideas or concepts* by *oneself* aiming to *actively bias one’s own perceptual, brain or interoceptive states, as well as the valence of perceived sensations*. *This reiteration takes a verbal/linguistic form (internally or out loud) and may be reinforced by employing imagery. Autosuggestion may take both forms: implicit (i.e., adopted and internalized suggestion from external sources) and explicit (applied consciously and volitionally).* Here we focus on the explicit (conscious) forms of autosuggestion set out for beneficial effects of the user. The word ‘actively’ indicates that autosuggestion is volitional and intentional, and links to concepts such as agency or free will (see below). Intention is directed towards a *predefined outcome, often contradictory to the existing experience,* to bias subsequent perceptual or brain states. This influence is assumed to be reflected at a phenomenological, behavioral, and neurophysiological level (see below).

Autosuggestion may also be regarded as a reactive form of a self-regulatory mechanism (i.e., ‘late correction mechanism’; Braver 2012), as opposed to a proactive form of cognitive control. Proactive versus reactive modes of cognitive control form the dual mechanisms of control (DMC) framework (Braver 2012). In a proactive mode of control, one acts by actively maintaining a goal-relevant information. This aims at ascertaining goal obtention in the case of cognitively demanding circumstances, which could jeopardize this goal achievement. For instance one might intend to go shopping right after work and thus keep this goal in mind throughout a working day to remember. This constant employment of attentional processes assures that the shopping is done (the goal is obtained, the beneficial effect), but it is also associated with cognitive costs. For example one may lack concentration on doing duties at work and one thus may make errors. In reactive control, on the other hand, attentional processes and goal representations are initiated in response to triggering events. This dependent character of reactive control mechanisms may fail in assuring goal obtention or maintenance, if the external cues are not salient enough. However, it allows allocating attentional cognitive reserves to performing tasks at hand. Referring to the above example, one should be very efficient at work (as the cognitive resources are all employed into performing the tasks at hand), but one would not be able to go shopping because the shops have already closed by the time one remembers the goal. In this framework, autosuggestion can be defined as a reactive form of cognitive control, because in autosuggestion, one tries to bias or override an *existing* perceptual state into a desired perceptual state. Possible costs inherent to autosuggestion processes may be reduced availability of cognitive resources to bias unwanted perceptual states or dependence on upcoming signals and insufficient conflict detection mechanisms, which may reduce the success of autosuggestion.

The question of whether or not one’s own mind has the capacity to influence one’s own perceptual and brain states has been debated by philosophers, psychologists, and neuroscientists for centuries (Fuchs 2006; Hegel and Inwood 2007; Maler 2017; Gregory and Zangwill 1987). We do not intend to re-awaken this debate here; rather, we aim at focusing on available experimental evidence from the field of cognitive neuroscience that provides us with empirical data on the factors that induce and limit the ability to control one’s own brain and perceptual states in an experimental setting. For example, it has been shown that placebo suggestions can modify functional activation and related pain thresholds at the level of the spinal cord through downstream projections (Eippert et al. 2009; Wright 1995), and neural activity often reflects inferred rather than actual brain states, for example via predictive coding (Kok and de Lange 2015, Friston 2012; Barron et al. 2020). Modern cognitive neuroscience provides empirical evidence that cognitive states that are believed, observed, or predicted can affect basic neurophysiological processes at the level of the spinal cord, subcortical structures (Sedley et al. 2016), or primary sensory cortices (Kuehn et al. 2018). These data form the basis for our concept and discussion on autosuggestion where our aim is to provide a conceptual overview over the shared and unique features of autosuggestion in relation to other phenomena.

We are aware that the question of whether top-down control influences perception itself, or only the interpretation of the perception, is an open and debated topic in psychology and philosophy. For instance, according to the concept of ‘cognitive penetrability’ (Pylyshyn 1980), one would assume that perception itself cannot be altered by autosuggestion, because perception is part of the cognitive architecture (and the cognitive architecture can by definition not be altered by beliefs and other forms of top-down control). However, whether perception itself or the interpretation of perception is altered by top-down control is a topic too multifactorial to be solved in the context of the present review. Rather, when we discuss the influence of autosuggestion on human thought and behavior, we will refer to the modulation of “states” in the context of this review. We will either refer to “brain states” in the case of neuroimaging, or to “perceptual states” or just “states” in the case of behavioral measures. With this, we aim at describing the phenomenon of investigation without explicitly commenting on the part of the cognitive architecture that is modulated from a conceptual point-of-view.

Box 1 Definitions

**Table:** 

Autosuggestion Instantiation and reiteration of ideas or concepts by oneself aiming to actively influence one’s own perceptual, brain or interoceptive states, as well as the valence of perceived sensations
Suggestion A thought or an idea that influences cognitive and physiological states
Heterosuggestion A process used by one individual to influence cognitive and physiological states of another individual through direct or indirect suggestion
Mental imagery A process of creating a mental representation of the object in absence of sensory input
Autogenic training A relaxation technique composed of multiple sub-parts aimed at facilitating desired bodily perceptions
Hypnotic suggestion The phenomenon where one individual gives a series of instructions to another individual aiming at modifying a range of subjective experiences and behaviors within a person being hypnotized
Implementation intentions A process of planning to respond to a certain situation in a specific way with the intention of assuring specific goal attainment
Reappraisal A process of changing an emotional response to a situation by thinking differently about the situation

### Empirical evidence on autosuggestion and related phenomena

The idea that suggestion can influence perceptual states is in accord with our everyday experiences. We can, for instance, instantly generate a feeling of hunger if we mistakenly believe it is lunchtime (Parkyn 1906), and thinking of itching suffices to raise the sensation of itching at a specific body part. Furthermore, the expectation or prediction of a future state can influence brain activity; incoming signals that are perceived as a surprise are, for example, weighted more than those that were already predicted (Weiss and Schütz-Bosbach 2012). However, which empirical evidence is available on autosuggestion?

We did an extensive search to identify scientific evidence on autosuggestion. We searched predominantly on Google Scholar, using the terms ‘autosuggestion’, ‘top-down control’, ‘self-suggestion’, ‘self-influence via thoughts’, and ‘self-regulation’ as search items. Our focus was mostly on the use of autosuggestion in the somatosensory context, but we also considered studies in other domains when relevant to identify cognitive mechanisms and neuronal correlates. In most cases, the experimental procedures did not introduce how they define autosuggestion nor did they formally distinguish between autosuggestion and other intervention techniques (e.g., Schlamann et al. 2010), but we developed precise criteria to distinguish one intervention from the other based on the experimental paradigms used (see Discussion).

Ludwig et al. (2014) investigated the effect of autosuggestion and posthypnotic suggestion on the value people place on unhealthy food during decision making. In the hypnosis group, individuals were suggested that a particular background color on the monitor would be associated with a feeling of disgust either towards sweet or salty snacks. In the autosuggestion group, individuals were required to make the same association by themselves. Both groups carried out an auction on the snacks, while fMRI measurements were taken. Both groups significantly reduced the amount of bidding assigned to the snacks associated with disgust. Moreover, both groups showed a decrease in blood oxygen level-dependent (BOLD) signal in the ventromedial prefrontal cortex (vmPFC), which is known to represent value, indicating reduced desire to eat those snacks. Yet, the depreciating effect of the cue on the rostral anterior cingulate cortex (rACC) was more pronounced in the hypnosis group as compared to the autosuggestion group. A weakness of this study is that there was no control group included without suggestive intervention, with both groups required to perform exactly the same association. It is therefore difficult to distinguish the effect of pure color-value association from the effect of the suggestive manipulation. Indeed, associations linking oneself (in this case, I, as the subject of feeling disgust) to an object has been shown to be fast, and without the need to reinforce any suggestion (Sui et al. 2009).

Autosuggestion is often used as a tool in therapeutic and relaxation methodologies, such as autogenic training (AT; Schultz 1973). Autosuggestion is implemented in autogenic training because the inner repetition of a thought or sentence is used to trigger somatic sensations (e.g., feeling of coolness on the forehead; Kanji 2000). However, the sentences used in AT do not always comply with linguistic guidelines (see Discussion).

An fMRI study by Schlamann and colleagues (2010) investigated brain activity during three autosuggestive phases of AT (being calm, the arm is heavy, and the arm is warm) in participants experienced in AT (AT-group) and participants who never practiced AT before (control group). The AT-group showed higher activation of the left pre- and postcentral cortices as compared to resting state, whereas the control group showed larger activation of the left parietal cortex and lower activations of the prefrontal and insular cortex as compared to the AT-group. Moreover, insular activation was correlated with the number of years of practice in simple relaxation techniques. This is an example of a study indicating that the concentration on sensations at specific body parts as induced by autosuggestive techniques can induce changes in brain networks that are related to top-down control and bodily awareness, particularly in people experienced in this technique. However, the actual effect of autosuggestion, as compared to other relaxation techniques, is unfortunately not tested here, as no other technique was compared against AT.

Autosuggestion is also part of the so-called cognitive behavior therapy intervention (CBT), which aims at alleviating symptoms via challenging and realigning maladaptive thoughts with reality (Longmore and Worrell 2007). One study investigated the effect of the CBT intervention on quality-of-life in geriatric patients (Sari et al. 2017). Participants were divided into autosuggestion and control groups. Participants in the autosuggestion group were asked to construct the autosuggestive phrases themselves according to their health preferences, and specific rules (for details see: Sari et al. 2017). Such constructed autosuggestive phrases were then recorded for participants in their own voice, and they were told to listen to these recordings a few times a day for the next 30 days. Both groups received their usual medical treatment. After the intervention, the autosuggestion group rated their quality of life higher and the serum cortisol level reached the healthy norms for elderly adults, as compared to the control group. This indicates that autosuggestion improves subjective experiences of quality of life and individual stress levels. However, the fact that the control group was not engaged in any other task, and that participants listened to the tapes rather than generated autosuggestion internally (see Box 1), makes it difficult to distinguish between related concepts such as attention or heterosuggestion as discussed below.

Taken together, the literature on autosuggestion measured explicitly is scarce (see Table 1). The studies implementing elements of autosuggestion support the claim that its use may have beneficial effects on people’s lives (e.g., restoring hormonal homeostasis). Furthermore, research suggests that the neuronal correlates of autosuggestion include prefrontal and insular cortices (Ludwig et al. 2014; Schlamann et al. 2010). There are, however, other cognitive processes that seem to share experimental parameters of autosuggestion, but have been related to different cognitive concepts, such as mental imagery (Anema et al. 2012), mental simulation (Jeannerod and Pacherie 2004) or bodily attention (Longo et al. 2009). These will be discussed in the next section in light of the concept of autosuggestion introduced above.

**Table.1:** Overview of studies assessing autosuggestion and related phenomena

Phenomena measured	Definitions	Sample size	Dependent variables	Control group	Methods	Results	References
Autosuggestion	Not given	*N* total = 60, aged ≥ 60, 30 per gr	Quality of life ratings Levels of serum cortisol concentration Levels of immunity markers	Yes	A tapes QoL chart Measurements in cortisol level and psycho-neuroendocrine immunology markers by magnetic resonance spectroscopy	Higher QoL scores in A group Serum cortisol reaching healthy norms in A gr. Increase in immunity markers in A group	Sari et al. (2017)
Posthypnotic suggestion, autosuggestion	‘Autosuggestion refers to the process of implementing a mental change in oneself (e.g. by repeating suggestions to oneself and by engaging in goal-directed imagery).’	*N* total = 32, 16 per gr	Number of bids for sweet/salty snacks	No	fMRI Questionnaire Behavioural: decision making	Snack devaluation by both H and A Effects stronger in hypnosis Decreased BOLD signal in the vmPFC	Ludwig et al. (2014)
First three autosuggestive phases of AT, motor imagery	Not given	*N* total = 38, 19 per gr	BOLD signal levels in experimental tasks and resting state	Yes	fMRI Questionnaires Motor imagery	Left parietal cortex activation during the first two steps of AT in contrast to resting state in controls Higher activation of prefrontal and insular cortices in AT group Higher activation in sensory-motor areas (*) during imagery task in AT group as compared to controls	Schlamann et al. (2010)
Verbal suggestion/placebo	‘Placebos—a set of ‘words, rituals, symbols and meanings’ that can change the brains of the patients’ (Benedetti et al. 2011)	*N* = 24, 14 in placebo-like gr, 10 in control gr	Amplitude measurements of late SEPs (N140 and P 200) before and after treatment	Yes	Electrical stimulation Baseline session, experimental manipulation and final recording	No increase of tactile sensation after the treatment No modification in late SEPs	Fiorio et al. (2014)
Positive and negative Suggestion/Placebo effects	Not given	*N* = 36, 13 per gr	Pain threshold, pain tolerance and pain endurance measures	Yes	Hand immersion in ice cold water	Higher pain thresholds, greater pain tolerance and greater pain endurance in PP gr compared to other groups	Staats et al. (1998)
Placebo analgesia effects	Not given	*N* = 24 (exp 1) *N* = 23 (exp 2)	BOLD signal on placebo analgesia PFC activation during pain anticipation Subjective pain ratings	Yes	fMRI Painful stimulation Placebo analgesia	Reduced activity in the thalamus, insula, and ACC after placebo analgesia Increased activity during anticipation of pain in PFC and midbrain Greater reported pain for control than placebo condition	Wager et al. (2004)
Autogenic training (AT), cognitive selfhypnosis (CSH)	Not given	*N* = 156, 58 outpatient neurological patients, 48 community members, 40 students	Treatment outcomes for chronic headaches Relations of level of hypnotizability to treatment outcome Subject recruitment on treatment outcome Use of analgesic medication	Yes	Pretreatment, post-treatment (week 8) and follow-up (week 35)	Reduction in HI scores in experimental groups compared to controls during treatment Reduction in HI scores in AT gr at post treatment differed sig. from WLC gr No sig. differences between treatment conditions at follow-up No sig. reduction in analgesics use between all groups	Ter Kulie et al. (1994)
Imagery	Not given	*N* = 40	Pressure pain thresholds	Yes	Pressure pain (induced by other, self, or other while imagining the pressure to be self-induced)	Elevated pain thresholds in self and imagery conditions (sig. differences between all conditions)	Lalouni et al. (2021)
Hypnosis	‘Ideomotor movement’—hypnotic phenomenon in which self-produced actions are attributed to an external source	*N* = 6 highly hypnotisable	Neural correlates of active movements correctly attributed to the self or misattributed to an external source	Yes	PET Hypnotic induction Deepening induction	Sig. higher activations in the PC in active movements attributed to an external source compared to identical movements attributed to the self	Blakemore et al. (2003)
Spiritual meditation, secular meditation, muscle relaxation	Not given	*N* = 83	Pain tolerance Headache frequency Mental and spiritual health variables	Yes	Cold pressor task	Greater decreases in the headache frequency in spiritual meditation gr compared to other groups Greater increases in pain tolerance, headacherelated self-efficacy, daily spiritual experiences and existential well-being in spiritual meditation gr compared to other groups	Wachholtz and Pargament (2005﻿)

*A* autosuggestion, *ACC* anterior cingulate cortex, *AT* autogenic training, *BOLD* blood oxygen level-dependent, *exp* experiment, *gr* group, *H* hypnosis, *HI* headache index, *NP* negative placebo, *PP* positive placebo, *PC* parietal cortex, *PET* Positron Emission Tomography, *PFC* prefrontal cortex, *vmPFC* ventromedial prefrontal cortex, SEPs somatosensory evoked potentials, *QoL* quality of life, *WLC* waiting list control

*AT: postcentral BA 7 and BA 5, sup. frontal BA 6, inf. parietal (BA 40); controls: postcentral BA 5, sup. frontal BA 6, inf. parietal (BA 40)

### Autosuggestion versus imagery, bodily attention and mental simulation

Mental imagery can be defined as a process of creating a mental representation of the object in the absence of sensory input (see Box 1). Several studies have shown that the neural correlates of the processes involved in mental imagery share similarities to the ones driven by the perception of the corresponding physical stimulus, but that they are often weaker in amplitude (Ganis et al. 2004; Kosslyn et al. 1997; Kuehn et al. 2014, 2018; Schmidt and Blankenburg 2019; Senden et al. 2019).

Similar to autosuggestion, mental imagery can be used to induce perceptual states. Fardo and colleagues (2015) showed that participants’ intensity perception of painful stimuli at the forearm was reduced when imagining a glove covering the forearm (pain inhibition condition), and increased when imagining a lesion (pain facilitation condition). These behavioral changes were correlated with modulations in pain-related potentials as measured with electroencephalography (EEG): in the pain inhibition condition, there was a rise in the amplitude of the N2 pain-related evoked potentials compared to baseline, whereas the reversed effect was reported in the pain facilitation condition. In this regard, mental imagery may lead to similar perceptual and neurophysiological outcomes as what is intended through autosuggestion (e.g., reduced pain), but the underlying cognitive mechanism may be different. Of note is that in Fardo and colleagues’ paper, the effects were due to imaging a glove to cover a lesion, but they were not due to trying to change the perceptual state itself. In a paradigm on autosuggestion, on the other hand, participants should be asked to directly modify specific perceptual states to predefined perceptual states. Both strategies may in part recruit different neuronal networks.

Both inhibitory and facilitatory mechanisms are likely involved in autosuggestion and mental imagery. Even if mental imagery’s content suppresses an ongoing experience (like decreasing pain perception while imagining a protective glove), this inhibition is usually a side effect of imagery. In the case of autosuggestion, what may potentially be inhibited is the non-desired perceptual state in order to facilitate the desired perceptual state. However, at this point, it is still unclear whether autosuggestion entails the suppression of an existing perceptual state and the creation of a novel perceptual state (perhaps involving different brain networks), or whether the existing perceptual state is biased and therefore “overwritten”. Facilitatory mechanisms should also play a role in mental imagery as well as autosuggestion. In both concepts, imagined or autosuggested states aim at inducing changes on a perceptual or brain level. Moreover, autosuggestion is an intentional process, where one tries to engage cognitive resources into creating desired results, expressed in the physical world. The facilitation of previously inhibited brain networks (i.e., disinhibition) or the activation of previously silent brain networks (i.e., facilitation) may therefore both contribute to successful autosuggestion.

Experimentally, mental imagery is often induced by providing participants with the to-be-imagined experience either before or in the course of the experiment, or by using everyday experiences everybody is familiar with, to later request their recall. In autosuggestion, on the other hand, experimental induction implies asking participants to modify a perceptual state of a certain feature towards a new perceptual state of that same feature. In this respect, there is a conceptual difference between asking participants to “remember the pleasant touch that you felt at the beginning of the experiment”, and asking them to imagine that “the touch that you will feel next feels pleasant” despite the touch feeling neutral or unpleasant.

Drawing attention to the body can change bodily states and their subsequent perception. For instance, just looking at the body can lead to an increase in its temperature (Sadibolova and Longo 2014). Also, visually attending to the body leads to analgesic effects when receiving painful stimulation (Longo et al. 2009). However, these effects are driven neither by imagining them to occur, nor by using controlled thoughts to achieve them; rather, these are implicit ‘*side-effects*’ of attending to the body, and do not fulfil the criterion of a volitional and intentional change in perception. In addition, there is no direction in the effect, as attention or looking at the body part lead to the same outcome (e.g., reduced tactile thresholds), with little control over the sensory perception. Autosuggestion should therefore be regarded in contrast to the aforementioned processes of imagination and attention, because in autosuggestion, one is attending to a tactile perceptual experience with the intention to modify it to the desired state, i.e., to decrease it (e.g., painful stimulation), or to increase it (e.g., pleasant affective touch). In both cases, however, attention is equally directed to touch.

In addition to mental imagery and attention, the concept of autosuggestion is also related to the concept of mental simulation. Both autosuggestion and mental simulation are dynamic processes, and they both lead to perceptual state changes. Mental simulations are considered forward-directed (Springer et al. 2013) and automatic (Markman et al. 2012), and they change with training (Decety and Ingvar 1990). Mental simulations may also be intentional when used as a specific term to describe a process similar to mental imagery (e.g., Ji et al. 2016), but that is not what we are referring to here. We discuss mental imagery in a separate paragraph. Mental simulations are often discussed under the umbrella of the forward model of motor control (Miall et al. 1993), according to which the sensory consequences of motor commands are compared to the actual consequences of movements (i.e., sensory reafference). Mental simulations of actions therefore precede the action that is observed or conducted. Deviations from expected and actual sensory perceptions are the basis for prediction errors, as postulated by predictive coding theory (Kok and de Lange 2015; Friston 2012; Barron et al. 2020). These prediction errors are corrected and minimized through learning (Ohlsson 1996). However, so far, not many cases are known where mental simulations are actively controlled by the participant.

Recently, the discrepancy between mental model updates that happen in the course of experience, and mental models (e.g., priors) that prevail in spite of contrary evidence has been pointed out (Lange et al. 2018). The authors noted that some sensory predictions are updated due to contrary evidence, and based on the reliability of stimuli and expectations, and therefore adapt to everyday experiences. Other predictions, however, are relatively constant throughout an individual lifetime, and might be more “rigid” against empirical counter-evidence. In this respect, research on autosuggestion seems to tap into this question: in which respect is successful autosuggestion dependent on the extent that priors can be changed? These forms of mental simulations are dynamic and future-directed, but usually require, similar to mental imagery, a reinstatement of previous experiences. Autosuggestion, in contrast, involves modification of ongoing (and possibly conflicting) sensory or motor perceptual states, and actively creating new predictions of desired outcomes.

Taken together, the concepts of autosuggestion, mental imagery, and mental simulation are related and may share important neural circuits and cognitive processes. All three require the recreation of representations regarding a state that is not currently perceived but rather created ‘offline’. Whereas in the case of mental imagery, these states are usually static and not necessarily future-directed, autosuggestive processes are dynamic and future-oriented. In cases where participants are asked to simulate non-performed movements, mental simulations mostly involve the reinstatement of a previous memory, and are usually not in conflict with a dynamic, ongoing input (Kent and Lamberts 2008). Autosuggestion is therefore unique as it requires access to a dynamic and future-oriented process that is not necessarily overlearned, and that may require changing or “overwriting” existing predictions.

### Autosuggestion versus implementation intentions and reappraisal

**Fig. 1:**
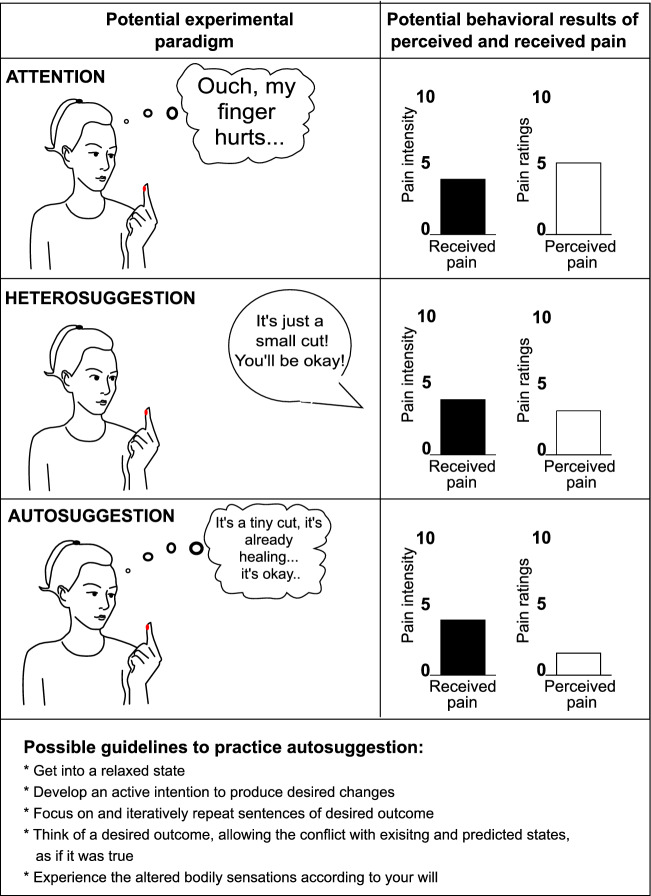
Conceptual representation of autosuggestion in a hypothetical comparison to attention and heterosuggestion in the context of a painful experience. Upper* panel*: The person directs attention to a painful experience at the finger. Ratings of perceived pain (white bars) are higher than the actual pain intensity (dark bars). *Middle panel*: The person experiences pain, and receives heterosuggestion from another person. Ratings of perceived level of pain are a bit lower than actual pain intensity. *Lower panel*: The person actively intends to reduce the perception of pain via autosuggestion. Ratings of perceived level of pain are significantly lower than actual pain intensity. *Fourth panel:* potential example guidelines to practice autosuggestion in an experimental setting

We wrote earlier that autosuggestion is a process driven by the intention of achieving a certain (brain or perceptual) state. It is in this respect relevant to distinguish autosuggestion from the concept of implementation intentions (Gollwitzer 1999). Whereas in both cases, there is a prespecified situational trigger leading to evoking different perceptual states, the two processes differ with respect to the time scales and strategic components. In autosuggestion, one reacts to an existing brain or perceptual state (for instance, the perception of pain at the fingertip, see Fig. 1) by aiming at biasing it into the desired state. The concept of implementation intentions assumes, however, that one plans to respond to a future specific situation in a predefined manner (i.e., one implements an intention of goal-directed responses when hurt at the fingertip; Gollwitzer 1999). Thus, whereas in implementation intentions, one aims at solving potential problems in translating one’s goals into action, in autosuggestion, one takes action to directly obtain the desired goal. In this way, in the process of learning to integrate autosuggestion into one’s everyday life, implementation intentions may be relevant.

Autosuggestion also relates to the concept of reappraisal. During appraisal, emotions are caused not by the situation itself, but by what the situation means to the perceiver with respect to related emotional concerns. By reappraising, one biases emotions by changing appraisals (i.e., the emotional impact of the situation). In other words, one can change an emotional response to a situation by thinking differently *about the situation* (Uusberg et al. 2019). This is related to but also different from autosuggestion. For example, if one experiences muscle pain after a workout in the gym, in autosuggestion, one would directly target the perceptual state of pain by reducing it, whereas in reappraisal, one has more choices of approaching the painful experience. One could change how one feels *about* the pain after exercising, for example by accepting the pain but reducing its effect on behavior, or by even welcoming the pain as part of a sportive experience. Instead of focusing on its unpleasantness and discomfort, one would try to change the emotional impact of the painful experience.

### Autosuggestion versus heterosuggestion and hypnosis

Hypnotic suggestion and heterosuggestion are phenomena that are related but also different from autosuggestion (see Box 1). In hypnotic suggestion and heterosuggestion, an external individual gives a series of instructions to the to-be-suggested person that aim at modifying subjective experiences and behaviors. In the last decades, many researchers have used hypnotic induction as a tool to modify top-down control processes (Nash and Barnier 2012; for a review). A critical difference between hypnosis and autosuggestion is the diminished intention and sense of control in hypnosis compared to autosuggestion. Diminished control over one’s own bodily states is also typical for hypnosis in cases where hypnosis is self-induced. Hypnotic states are generally accompanied by feelings of involuntariness and a disrupted sense of agency (participant lacks the control over one’s own actions; Polito et al. 2015). For instance, Blakemore and colleagues (2003) used hypnosis to create delusions of alien control in healthy participants. In the study, participants underwent 4 conditions during the hypnotic state: 1. Active Movement (participant actively lifted his/her arm), 2. Real Passive Movement (the experimenter lifted participant’s arm via a pulley attached to the wrist), 3. Deluded Passive Movement (participant was told that his/her arm would be lifted by a pulley, but the pulley was not used), and 4. Rest Condition. Despite generating movements themselves, highly suggestible participants described the raising of their arm as being involuntary and without intention in the deluded passive movement condition. Conversely, participants correctly attributed the movements to themselves in the active movement condition. Using positron emission tomography (PET) imaging, significantly greater activations in the cerebellum and parietal operculum in the deluded passive movement as compared to the active movement condition were found. This is interesting, as the parietal operculum is known to be more active during passive as compared to active movements (Mima et al. 1999). These results demonstrate that identical active movements are processed differently in the brain depending on the attribution of these movements to oneself or to other people.

To those who like to be in control, this subjective feeling of self-control but also the actual ability to control oneself are major advantages of autosuggestion compared to hypnosis and heterosuggestion. It is thus clinically and practically relevant to determine whether attempts to implement a mental change by oneself are as effective, or even more effective, than attempts to implement a mental change by another person.

### Autosuggestion versus placebo

Placebo comprises a special type of heterosuggestion, which is not only transmitted by the other person, but also via the situational and social context (Miller and Kaptchuk 2008), and the prior experience of the individual (Colloca and Benedetti 2006). Placebo can be defined as an inactive treatment and/or a situational component of that treatment administered on a person to alleviate experienced symptoms or illness (Shapiro and Shapiro 1997). Externally evoked expectations (i.e., specific cognitions about the probability of future events; Rief et al. 2015), and consequently formed beliefs regarding the effects of treatment are factors strongly predicting the success of placebo responses (Beauregard 2007). These expectations can be triggered by external cues (e.g., white coat, the syringe; Petrie and Rief 2019), and can be strengthened by the inner desire for relief (Tracey 2010). The expectations of a specific cue leading to a specific outcome have been learnt (Petrie and Rief 2019), and they go beyond the belief of whether the treatment will work or not. Peoples’ expectations are also related to attributed values to a given treatment (e.g., a red, more expensive pill is more effective than the blue, cheaper one; Tracey 2010). Placebo responses are complex phenomena driven not only by people’s expectations and beliefs but are mediated also by personality and psychological traits (Tracey 2010). The belief in one’s own abilities to influence events in one’s own life are elements also constituting the concept of self-efficacy pinpointed by Bandura (Bandura 2010). Only if people believe in their capabilities in achieving certain goals (e.g., in the case of autosuggestion: evoking desired perceptual or brain states via autosuggestion), will they have the motivation to do necessary action (here: employing cognitive resources underlying autosuggestion). To date, the extent to which these play a role in autosuggestion is still to be addressed.

Most placebo research has focused on placebo responses to painful stimulation (e.g., Montgomery and Kirsch 1996; Petrovic 2002). For example, Wager et al. (2004) investigated the response to a placebo cream that is supposed to reduce painful sensations at the wrist. In the baseline condition, participants received intense and mild shocks, and were asked to rate the shock intensity. In the placebo condition, placebo cream was applied to participants’ wrists and half of the group was told that it would reduce but not stop the experience of pain (placebo group), while the other half was told that the cream was not effective in alleviating pain (control group). These instructions were reversed after half of the blocks, so that each participant belonged to both the control and experimental groups. Pain intensity ratings of intense shocks were significantly higher in the control group compared to the placebo group, while no differences were observed for mild shocks. Interestingly, the magnitude of the reported pain reduction between control and placebo conditions correlated with the magnitude of the reduction during shock delivery of fMRI activation in brain areas activated during pain processing. Moreover, placebo analgesia was coupled with increased activation in prefrontal brain areas. These results indicate that external cues, here a placebo cream, are effective in influencing cognitive, sensory, and affective pain perception.

It is relevant to ask whether one of the mechanisms inducing the placebo response could be explained by autosuggestion (Jakovljevic 2014). In that view, ideas, once presented by the professional, are believed and become internalized by the patient, and may be further internally reiterated and acted upon accordingly. This reinforcement of received suggestions, coupled with formed expectations and beliefs in the success of the treatment, could be a starting point for autosuggestion. For example, Staats and colleagues (1998) conducted an experiment on pain perception that is related to both autosuggestion and placebo. The task was to keep the dominant hand in iced water (approximately 1 °C) for as long as possible or until experiencing pain. Participants received instructions and suggestions about the effects of immersing the hand in cold water, which could be positive (positive placebo group), or negative (negative placebo group), and were asked to iterate given information during hand immersion. Participants in the control group were informed about the goal of the study and that they should think of nothing particular during hand dipping. The positive placebo group showed higher pain thresholds, greater pain tolerance, and greater pain endurance as compared to a first immersion without instruction, and as compared to other groups during second hand immersion. Moreover, participants’ levels of anxiety and worry significantly decreased, and they showed significant increases in the self-reported ability to cope with pain. The negative placebo group showed the opposite results. The process of re-stating the received suggestion and repeating it ‘covertly’ could therefore reflect the process of autosuggestion, even though the authors here refer to it as “placebo”. However, the effect of *reiteration* of the given suggestion was not isolated in this study from the effect of *receiving* a specific suggestion, thus further research is needed to experimentally disentangle these two effects from each other.

## Discussion and future directions of autosuggestion research

Driven by existing evidence and by gaps in the literature, here we aimed to develop a working definition of autosuggestion. We define autosuggestion as the *instantiation and reiteration of ideas or concepts* by *oneself* aiming to *actively bias one’s own perceptual, brain or interoceptive states, as well as the valence of perceived sensations*. *This reiteration takes a verbal/linguistic form (internally or out loud) and may be reinforced by employing imagery. Autosuggestion may take both forms: implicit and explicit.* We assume that other cognitive strategies may be implemented in the process of autosuggestion, such as mental imagery or mental simulation, which may, however, be controlled for in a carefully designed study. This definition allows differentiating the process of autosuggestion from other phenomena such as heterosuggestion, hypnosis, mental imagery, mental simulation (see Box 1).

The importance of top-down control in the form of autosuggestion and the great value of experiments investigating this phenomenon as asserted by Coué has been recognized (e.g., Ludwig et al. 2014; Paulhus 1993). Nevertheless, based on current data, we conclude that there is little empirical evidence on autosuggestion particularly when one wants to understand the relationship between autosuggestion and related phenomena. Thus, the answer to the question: ‘What are neural correlates underlying autosuggestion?’, remains to a large extent unclear.

We reviewed available evidence on autosuggestion and evaluated the methodology used in the experimental designs in reference to related concepts such as mental imagery, mental simulation, hypnosis, and placebo. We identified brain networks that could in principle be altered by autosuggestion including sensory cortices, the insula, but also cognitive control networks, and discussed the flaws in some experimental designs that were lacking appropriate control conditions or failed to disentangle autosuggestion from attention or placebo.

**Fig. 2:**
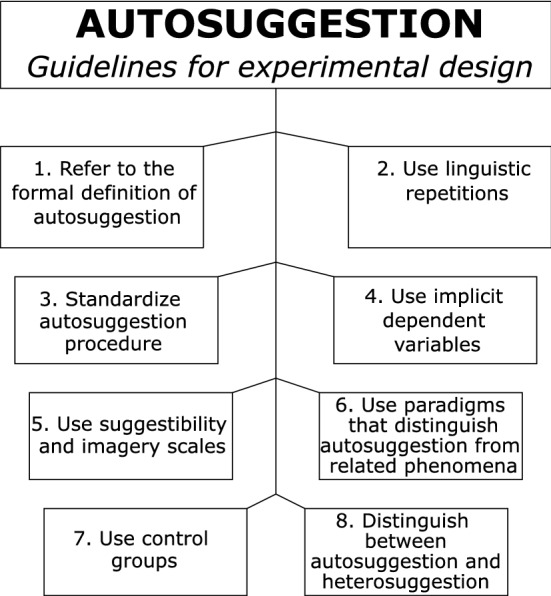
Directions for experiments on autosuggestion (for detailed description see in the text below)

We will now take this evidence to propose an alternative approach to examine cognitive and neural mechanisms of autosuggestion in an experimental setting (see Fig. 2).

Here, based on the concepts and experimental approaches outlined above, we provide eight recommendations that, in our view, would be beneficial to follow when designing or implementing an autosuggestion condition into an experimental setting. We do not claim that these recommendations are all inclusive, because future experimental approaches or results may require adjustments or refinements. Nor do we claim that these recommendations are generally applicable, since specific experimental designs or participant groups may require individualized treatments or experimental setups. Rather, we here aim at providing a first overview over potentially necessary steps and preparations needed in order to successfully design an autosuggestion experiment.

First, any experimental approach that aims to investigate autosuggestion should provide and follow a formal definition of autosuggestion (Fig. 2, Point 1). This helps to communicate between researchers and readers which phenomenon is examined, and also allows a comparison of the effectiveness of different concepts and approaches.

Second, any experimental approach that aims to investigate autosuggestion should include a linguistic repetition of to-be-experienced states to be followed by the to-be-suggested person (Fig. 2, Point 2). This linguistic repetition can be spoken out aloud or repeated internally. The linguistic constructions should motivate to modify current experiences and should (1) refer to ‘I’, (2) state the desired outcome in the present tense, as if it were already true, (3) be short and concise, and (4) be stated in positive terms (i.e., avoiding negations, e.g., ‘I am okay’ rather than ‘I am not in pain’).

Third, the procedure of inducing autosuggestion in an experimental setting should be standardized (Fig. 1, Bottom panel) to reduce variability and increase homogeneity in the results. Thus, structured rather than ‘free’ autosuggestion should be increasingly used in future experiments (Fig. 2, Point 3). By structured, we mean the use of the same type of linguistic repetition and autosuggestion procedure (e.g., including or not imagery, using a predetermined statement) across participants.

Fourth, the dependent variable used in the experimental design to measure autosuggestion should be implicit (i.e., the brain or physiological state that is used as a dependent variable is different from the brain or physiological state participants are ‘autosuggesting’; Fig. 2, Point 4). This is critical, because otherwise the process of autosuggestion cannot be disentangled from compliance of the participants towards the experimenter (i.e., demand characteristics; Orne 1962). If this is not entirely possible, there should be at least measures that cannot be voluntarily modulated by the participant, such as the recording of physiological correlates without providing feedback (e.g., EEG, fMRI).

Fifth, suggestibility scales (e.g., Multidimensional Iowa Suggestibility Scale; Kotov et al. 2004) and mental imagery scales (e.g., VVIQ; Marks 1973) should be used in parallel when investigating autosuggestion (Fig. 2, Point 5). The use of these scales helps to investigate the generalizability and necessary skills needed to successfully practice different forms of autosuggestion (e.g., high suggestibility, high abilities in mental imagery), and sheds more light on common underlying mechanisms. It also helps to decide which individuals may be suitable to be included in autosuggestion interventions in clinical settings. Moreover, ratings related to beliefs in the ability to successfully obtain the desired results via autosuggestion should be taken in order to understand the contribution of one’s own belief on the effectiveness of autosuggestion.

Sixth, experiments should aim at distinguishing between autosuggestion, attention, and mental simulation (Fig. 2, Point 6). To control for attention, one possibility is to flexibly modify the direction of the effect in different conditions, because attention usually modulates the direction of the attended sensation towards enhancement. To control for mental simulation, conflicts between existing and future states can be created, because autosuggestion should allow the modulation of conflicting future states.

Seventh, one or multiple adequate control groups or control conditions need to be implemented into the experimental design in order to assure that observations are caused by the manipulation itself, and not by other factors (e.g., training effects, time; Makin and Xivry 2019). Taking care of appropriate experimental control conditions (e.g., autosuggestion vs imagery) can elucidate which of these techniques are more successful to trigger a certain experience (Fig. 2, Point 7), which is also critical information for clinical interventions.

Eighth, any experimental approach that aims to investigate autosuggestion should clarify whether autosuggestion or heterosuggestion is investigated (Fig. 2, Point 8). That is, it is important to clarify which agent induces the change (participant or experimenter). If written instructions on screen are used, it should be clarified before whether these instructions are perceived by the participant as their own instructions (for example if they can choose their own linguistic repetition, or if they incorporated the instructions as their own), or if they are perceived as instructions by the experimenter to the participant. Such small modifications can change the results of the experiment, and may involve different cognitive mechanisms. In particular, the influence of the experimenter is expected to be much higher in heterosuggestion compared to autosuggestion.

To our knowledge this is the first comprehensive review about the cognitive phenomenon of autosuggestion. The combination of findings provides support for conceptual premises that top-down mechanisms, such as autosuggestion and related phenomena (e.g., placebo (Blair 1965), autogenic training (Kanji 2000), imagery (Fardo et al. 2015)), can effectively create changes within the body on a behavioral, cognitive and neural level. Despite its great potential benefits, autosuggestion has gained too little rigorous scientific interest so far. Thus, we wrote a list of outstanding questions regarding the phenomenon of autosuggestion listed in Box 2. Given the complementary benefits of the use of autosuggestion to other existing approaches, such as independence of a second person and having control over oneself, future investigations with more rigorous methodologies on this topic are urgently needed. If positive effects of autosuggestion can be proven scientifically, a new field of self-directed therapies may develop in clinical, therapeutic, and self-optimization settings.

Box 2 Open questions on autosuggestion

**Table:** 

Which brain networks are specifically involved in autosuggestion compared to other related phenomena? Are primary sensory areas involved in the process?
Are loud or internally reiterated linguistic repetitions more (or less) effective to induce autosuggestion?
Does mental imagery influence autosuggestion? Is autosuggestion possible without “believing” in it?
Do expectations and beliefs regarding one’s own capabilities in performing autosuggestion influence autosuggestion?
Which individual traits determine autosuggestibility? Is success in autosuggestion related to high levels of hypnotizability and imagery skills?
Can autosuggestion training reduce the time a person needs to induce autosuggestion? Can people who are not successful in inducing autosuggestion learn to be effective?
Can autosuggestion be an effective treatment for physiological or psychological disorders? And if so, how can we find out which individuals are particularly suitable for this?

## Data Availability

Data sharing is not applicable to this article as no datasets were generated or analysed during the current study.
